# The juji-gatame technique can be used in the special judo fitness test to evaluate fitness condition in grappling fighters: A crossover trial

**DOI:** 10.1097/MD.0000000000044255

**Published:** 2026-01-09

**Authors:** Paulo Francisco de Almeida-Neto, Alexandre Bulhões-Correia, Gilmara Gomes de Assis, Halil İbrahim Ceylan, Roberto Felipe Câmara Rocha, Iago Medeiros da Silva, José Carlos Gomes da Silva, Paulo Moreira Silva Dantas, Valentina Stefanica, Breno Guilherme de Araújo Tinôco Cabral

**Affiliations:** a Health Sciences Center, Federal University of Rio Grande do Norte, CCS-UFRN, Natal, Brazil; b Department of Physical Education, Federal University of Rio Grande do Norte, DEF-UFRN, Natal, Brazil; c Escola Superior Desporto e Lazer, Instituto Politécnico de Viana do Castelo, Rua Escola Industrial e Comercial de Nun’Álvares, Viana do Castelo, Portugal; d Sport Physical Activity and Health Research and Innovation Center, Viana do Castelo, Portugal; e Physical Education and Sports Teaching Department, Kazim Karabekir Faculty of Education, Ataturk University, Erzurum, Turkey; f Department of Physical Education and Sport, Faculty of Sciences, Physical Education and Informatics, National University of Science and Technology Politehnica Bucharest, Pitesti University Center, Pitesti, Romania.

**Keywords:** anaerobic capacity, combat sports, jiu-jitsu, sport, wrestling

## Abstract

**Background:**

The special judo fitness test (SJFT) is a widely used tool for assessing physical fitness in grappling athletes. However, its conventional format, centered on the ippon-seoi-nage throwing technique, may not adequately evaluate athletes specializing in ground-fighting techniques. Incorporating a ground-based technique, such as juji-gatame (JG), could enhance the test’s applicability for diverse grappling disciplines. To evaluate the feasibility and reliability of using the JG instead of the ippon-seoi-nage in the SJFT to assess physical fitness in grappling athletes.

**Methods:**

A randomized crossover trial was conducted with 150 male grappling athletes, including 94 juniors (13.4 ± 2.3 years; categorized by Tanner stages: 17 prepubertal, 48 pubertal, and 28 postpubertal) and 56 adults (30.3 ± 9.7 years). Participants performed both the conventional SJFT (using ippon-seoi-nage) and the adapted SJFT-JG (using juji-gatame) in a randomized order, with a 24-hour washout period between tests. Performance was assessed using the SJFT index and reliability metrics. Seven days later, a retest was conducted for both versions of the SJFT, again with a 24-hour interval between tests. It is important to note that the adapted version of the SJFT was performed using a training dummy.

**Results:**

The SJFT-JG demonstrated strong absolute (ICC = 0.99, LoA = -8.7; 7.8) and relative reliability (ICC = 0.98, LoA = -0.18; 0.16). No significant differences were observed in SJFT index scores between the 2 versions (*P* >.05), indicating physiological equivalence.

**Conclusion:**

JG can be reliably integrated into the SJFT as an alternative to ippon-seoi-nage, broadening the test’s applicability for grappling athletes, particularly those emphasizing ground techniques. This adaptation provides a practical and valid method for evaluating physical fitness across diverse grappling styles.

## 1. Introduction

The special judo fitness test (SJFT) is a fitness test developed for judo athletes^[1,2]^ in which the athletes must throw 2 partners of similar body mass and positioned 6 meters apart as many times as possible during the periods of 15, 30, and 30 seconds with 10-second intervals, using the ippon-seoi-nage technique.^[3]^ Alternatively, the test can be performed using training dummies when ideal partners are unavailable.^[4]^ The athlete’s heart rate (HR) is measured immediately after test HR_0_, and again 1 minute later HR_1_^[3]^; then, based on the number of correct throws and HR data, a fitness index is calculated, and the athlete’s fitness is classified in categories such as excellent, good, or poor.^[3–5]^

A recent study analyzed the reliability of the SJFT parameters (throws, HR, and index based on HR) through test/retest methods. The results indicated that the SJFT index based on HR shows better reliability and low measurement error (Cronbach’s α = 0.807, Error = 4.5%) for assessing fitness and performance in fighters.^[2]^ Considering that a judo match has an effort/rest ratio of 2:1 to 3:1 (20–30 seconds of effort for ~10–15 seconds of rest) and the SJFT was designed to simulate a 3:1 ratio.^[1]^ This characteristic allows the SJFT to be applied across different grappling combat sports that involve similar effort/recovery ratios, such as sambo and freestyle and Greco-Roman wrestling, among others.^[6–8]^

Grappling sports encompass a wide range of techniques, including throws, joint locks, and chokes. While the ippon-seoi-nage throw is widely used in judo and also appears in other disciplines such as mixed martial arts, sambo, and Brazilian jiu-jitsu,^[8,9]^ fitness in grappling is not exclusively developed or expressed through throwing techniques. Ground combat – especially the application of submissions like chokes or joint locks – plays a crucial role in the tactical structure of many grappling athletes, particularly in disciplines like jiu-jitsu and mixed martial arts.^[8]^ Therefore, evaluating physical fitness using techniques that reflect an athlete’s dominant tactical approach may offer more meaningful and sport-specific insights.

In this context, it is relevant to develop alternatives to the traditional SJFT protocol that incorporate ground techniques. A pilot study involving elite judo athletes demonstrated that replacing the ippon-seoi-nage with a preferred throwing technique did not affect the SJFT index, suggesting that the test’s validity may be preserved despite variations in technique.^[8,9]^ Building on this idea, the inclusion of a ground technique, such as the juji-gatame (JG) (an armlock), may further expand the test’s applicability to grapplers who specialize in ground fighting.

The JG is one of the most commonly applied submission techniques in grappling sports.^[8,9]^ It involves the attacker positioning themselves beside or above the opponent’s torso, isolating an arm between their legs while applying pressure to the elbow joint to induce submission. Given its frequency of use and biomechanical complexity, the JG also imposes meaningful physical demands that may serve as a basis for assessing combat fitness related to ground techniques.

Therefore, it is reasonable to hypothesize that incorporating the JG into the SJFT protocol (SJFT-JG) may produce similar performance outcomes to the classical version using the ippon-seoi-nage. This adaptation could provide grapplers with a more specific and ecologically valid method for fitness evaluation, especially for athletes whose competitive strategy relies predominantly on ground combat.

Accordingly, the aim of the present study was to examine the differences in performance outcomes between the traditional SJFT (using ippon-seoi-nage) and an adapted version employing the JG technique (SJFT-JG).

## 2. Methods

### 2.1. Study’s design

This trial crossover involved 150 male grappling athletes, including 94 juniors (17-prepubertal, 48-pubertal, and 28-post-pubertal, as determined by the Tanner stages clipboard^[10,11]^) and 56-adults. The minimum sample size was determined a priori using G*Power software (version 3.1, Düsseldorf, Germany).^[12]^ The calculation was based on the “F” statistic, with a standard alpha of 0.05 and a standard beta of 0.90, resulting in a recommended minimum sample size of 130 individuals (Power: 0.90).

Participants were recruited through social media, and the assessments outlined in this study were conducted in the biodynamics laboratory of the Federal University of Rio Grande do Norte, Brazil. The inclusion criteria required participants to be at least 7 years old and to have practiced a grappling sport (e.g., jiu-jitsu, wrestling, sambo, judo, etc) for a minimum of 1 year before the study (from beginner [white belt] to advanced [black belt] level). The exclusion criteria included having musculoskeletal injuries for at least 3 months and using exogenous substances that could affect anaerobic performance (e.g., creatine, betaine, or other supplements).

### 2.2. Ethics

This study was submitted and approved by the Ethics Committee of the Federal University of Rio Grande do Norte, Brazil (#29252120700005537) and followed the norms of the Declaration of Helsinki.^[13]^ The design of the present study was publicly available a priori on the Open Science Framework Registries platform (DOI: 10.17605/OSF.IO/TGP94). All participants (minors and adults) and their respective guardians (in the case of minors) were introduced to all research procedures, and those who agreed to participate in the research signed the informed consent form.

### 2.3. Procedures

**Figure 1.**
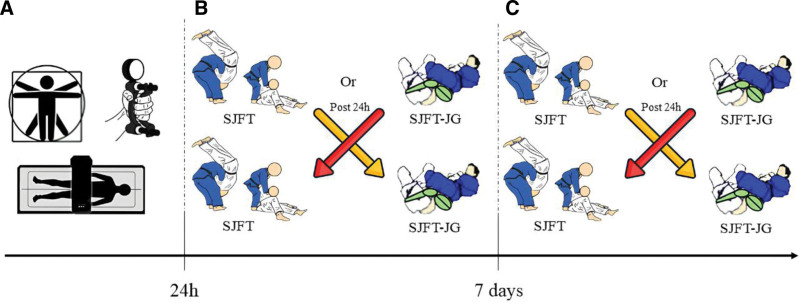
Study design. (A) Anthropometric evaluations (weight, height, body composition by dual-energy X-ray absorptiometry) and handgrip evaluation. (B) Randomization for the order of performing the conventional SJFT and the adapted version using the juji-gatame technique (SJFT-JG). Subsequently, a crossover was applied 24 h after the first testing phase. (C) Repetition of the procedures described in point “B” to conduct retest analyses. The analyses of point “C” were performed 7 days after the completion of the analyses of point “B.” SJFT-JG = SJFT using the juji-gatame technique.

Initially, the sample was characterized by collecting anthropometric and handgrip strength data. Within 48 hours, both versions of the SJFT (conventional and adapted) were performed, with a 24-hour interval between tests. Seven days later, a retest was conducted for both versions of the SJFT, again with a 24-hour interval between tests (see Fig. 1). It is important to note that the adapted version of the SJFT was performed with a training dummy, which ensured that participants did not have their performance limited for fear of injuring a real training partner.

### 2.4. Randomization

An external research collaborator performed randomization of the order of the fitness tests using the SJFT and the SJFT-JG, so participants did not know which test they would perform first.

### 2.5. Blinding

The tests were applied blindly by secondary evaluators (the main researcher did not participate in the application of the tests), and the statistical data analysis was performed blindly by an external collaborator to the research.

### 2.6. Sample characterization

The anthropometric data was assessed as follows: With participants barefoot and wearing light clothing, body mass was measured using a Filizola® digital scale with a capacity of 150 kg and a variation of 0.10 kg (São Paulo, Brazil). Height was determined by a Sanny® stadiometer (precision of 0.1 mm) (São Paulo, Brazil). For these procedures, we used the protocols of the International Society for the Advancement of Kinanthropometry (ISAK).^[14]^ All evaluations were performed by a single examiner, and for the intraobserver technical error of anthropometric measures, we adopted ≤1.0% as an acceptable margin.^[15]^ Lean and fat mass levels were analyzed by dual-energy X-ray absorptiometry (DXA) (LUNAR®/GE PRODIGY – LNR 41,990, Washington. Software enCORE, GE Healthcare®, version 15.0, Madison). For Junior fighters, appropriate algorithms for the pediatric population were used. DXA used the following standardization during the evaluations: Full Body Evaluation, Voltage (kV): 76.0, Current (mA): 0.150, Radiation dose (µGγ): 0.4 (Very low, no health risk).

In participants Juniors, the state of biological maturation was assessed as a biological age in years from the attainment of peak height velocity (PHV), termed maturity offset, where 0.0 years equal PHV; a measure of somatic maturation. Years from PHV was predicted using mathematical models proposed by Moore et al.^[16]^: Maturity offset in males = −7.999994 + [0.0036124 × (Age _(years)_ × Stature _(cm)_)]. Participants were classified as pre-PHV (maturity offset <−1.0 years from PHV), circum-PHV (between −1.0 and 1.0 years from PHV), and post-PHV (>1.0 years from PHV).^[16]^

Puberty was assessed using the Tanner stages clipboard to determine the stages of genital hair development.^[10,11]^ A nursing professional with extensive experience presented Tanner clipboards to participants individually in a private room. In this context, the importance of the examination was explained, along with the need for participants to provide truthful answers regarding genital hairiness. Subsequently, participants were given 5 minutes alone in the room to conduct a self-assessment. Upon completing this procedure, participants indicated the corresponding stage of hairiness on Tanner clipboard. Thus, the sample was classified as prepubertal (stage 1 of hair development), pubertal (stages 2, 3, and 4 of hair development), and post-pubertal (stage 5 of hair development).

### 2.7. Strength

The handgrip performance of the participants was evaluated using a hydraulic dynamometer (JAMAR®, Cambuci, Brazil).^[17]^ For this, participants were seated on a bench adjusted to their height and holding the dynamometer with their elbow flexed at a 90° angle. Three maximum grip contractions (3-s with 60-s recovery periods) were performed with both hands. The average of the best performance of both limbs was considered for sample characterization.

### 2.8. Special judo fitness test

**Figure 2.**
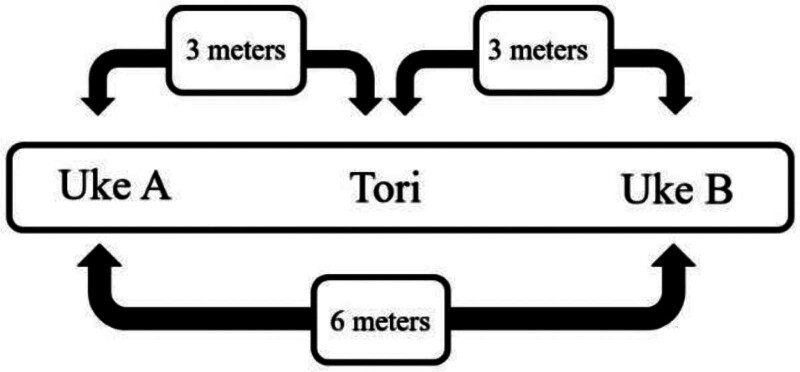
Illustration of the SJFT setup. SJFT = special judo fitness test.

The SJFT was performed with the athlete (Tori) executing the ippon-seoi-nage throwing technique alternately on 2 training partners (Ukes) with similar weight to their own, positioned 6 meters apart (Fig. 2), in 3 consecutive phases: 15 seconds (Phase A), 30 seconds (Phase B), and 30 seconds (Phase C), without intervals.^[1,2]^ During the test, athletes used a portable HR monitor, Polar® Strap (Model H10, Kempele, Finland) (short-range telemetry). HR was recorded immediately after the effort and 1 minute into recovery for subsequent analysis of the physical fitness index.

### 2.9. Adaptation of SJFT

**Figure 3.**
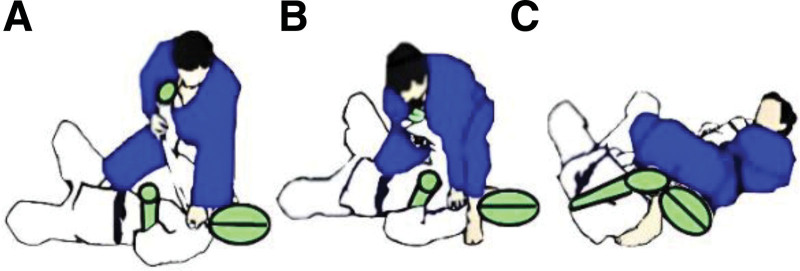
Implementation of the adaptation of the special physical fitness test for judo called special physical fitness test for judo using the juji-gatame technique (SJFT-JG). (A) The participant moves from the “mount” position and chooses an arm to attack. (B) The participant moves the foot positioned next to the dummy’s shoulder to the front of the dummy’s head. (C) The participant completes the application of juji-gatame.

In the adapted SJFT, named SJFT-JG (SJFT-JG), athletes used the groundwork technique JG from the “mounted” position on a training dummy (25 kg, 170 cm, 3Xtreme Sports®, São Paulo, Brazil) (Fig. 3). The training dummy was chosen to standardize the test application and optimize the space and safety of participants.

During the execution of the SJFT-JG, a single training dummy was used. To perform the test, the athlete was required to apply the JG technique starting with the dummy’s right arm, then return to the mounted position and apply the same technique to the left arm. This sequence was maintained throughout the test according to the prescribed time intervals and structure.

### 2.10. *Absolute and relative* physical fitness index

The HR was monitored for analysis of the physical fitness index via short-range radio telemetry using a Polar® strap (Model H10, Kempele, Finland) reinforced with adhesive tape to ensure proper fixation on the participant’s chest. The HR was recorded immediately after and 60 seconds after the tests. For both the SJFT and SJFT-JG, we calculated the physical fitness index of absolute anaerobic index using the equation: [(HR immediately after the test + HR 60-s after the test)/ number of executed techniques]. The relative anaerobic index was calculated by dividing the absolute anaerobic index of each test by the participant’s body mass, according to the recommendations of Isik et al.^[18]^

### 2.11. Statistical analyses

Data normality was tested using the Kolmogorov–Smirnov test, skewness, and kurtosis Z-score (-1.96 to 1.96) and was visually inspected using the QQ-Line plot. Concordance between methods regarding the moments of the present study (test and retest) was analyzed by Bland-Altman plot, adopting limits of agreement (LoA) values between −5 and 5 as acceptable.^[19]^ The intra-class correlation coefficient (ICC) was used to analyze the reproducibility between methods, interpreted by magnitude^[20]^: Absence: <0; Poor: 0 to 0.19; Weak: 0.20 to 0.39; Moderate: 0.30 to 0.59; Substantial: 0.60 to 0.79; Almost complete: ≥0.80. Comparisons between fitness index were performed using a repeated measures ANOVA test (measures: test and retest/ SJFT and SJFT-JG). The covariates age, PHV, Tanner stages, training time, and belt color were included in the ANOVA models to assess their potential influence on the results. The Bonferroni post hoc test was used to confirm significant differences identified by the ANOVA models. Effect size was verified by partial eta-squared (η^2^p), interpreted by magnitude^[21]^: small (<0.01), medium (between 0.02 and 0.06), and large (>0.14). All analyses and graphs were performed using GraphPad Prism software (Version 8.01 (244), California), considering *P* <.05 as the significance threshold.

## 3. Results

The sample characterization is shown in Table 1. Of note, there were no medical complications during the tests proposed in this study.

**Table 1:** Sample characterization.

Variables	Juniors (n = 94)	Adults (n = 56)
Chronological age (yr)	13.4 ± 2.3	30.3 ± 9.7
Tanner stage (pubic hair)	3.37 ± 1.5	–
Biological maturity (PHV)	−0.9 ± 1.5	–
Stature (cm)	161.2 ± 14.0	178.0 ± 6.9
Body mass (kg)	52.1 ± 15.4	81.5 ± 7.5
Lean mass (kg)	39.1 ± 12.2	67.9 ± 7.0
Fat Mass (kg)	11.3 ± 5.5	13.4 ± 4.9
Handgrip (kgf)	27.3 ± 10.7	45.4 ± 7.9
Training time (yr)	2.9 ± 1.9	11.2 ± 10.1
Weekly training load (d)	3.4 ± 1.3	4.5 ± 1.1
Daily training load (h)	1.0 ± 0.2	1.8 ± 0.9
Index SJFT – test (Beats·min^-1^·throw^-1^)	18.1 ± 3.7	13.7 ± 0.7
Index SJFT – retest (Beats·min^-1^·throw^-1^)	17.4 ± 2.9	13.3 ± 0.9
Index SJFT – test (Beats·min^-1^·throw^-1^·kg)	0.4 ± 0.2	0.2 ± 0.0
Index SJFT– retest (Beats·min^-1^·throw^-1^·kg)	0.4 ± 0.1	0.2 ± 0.1
Index SJFT-JG – test (Beats·min^-1^·throw^-1^)	19.1 ± 5.0	13.1 ± 0.3
Index SJFT-JG – retest (Beats·min^-1^·throw^-1^)	18.9 ± 5.2	13.0 ± 0.3
Index SJFT-JG – test (Beats·min^-1^·throw^-1^·kg)	0.4 ± 0.2	0.2 ± 0.1
Index SJFT-JG – retest (Beats·min^-1^·throw^-1^·kg)	0.4 ± 0.1	0.2 ± 0.0

Data processing: descriptive statistics.

Beats·min^-1^·throw^-1^ = beats per minute/number of throw, Beats·min^-1^·throw^-1^·kg = beats per minute/number of throw/kilogram, cm = centimeter, kg = kilogram, Kgf = kilogram force, n = absolute number, PHV = peak height velocity, SJFT = special judo fit test, SJFT-JG = special judo fitness test using the juji-gatame technique.

**Figure 4.**
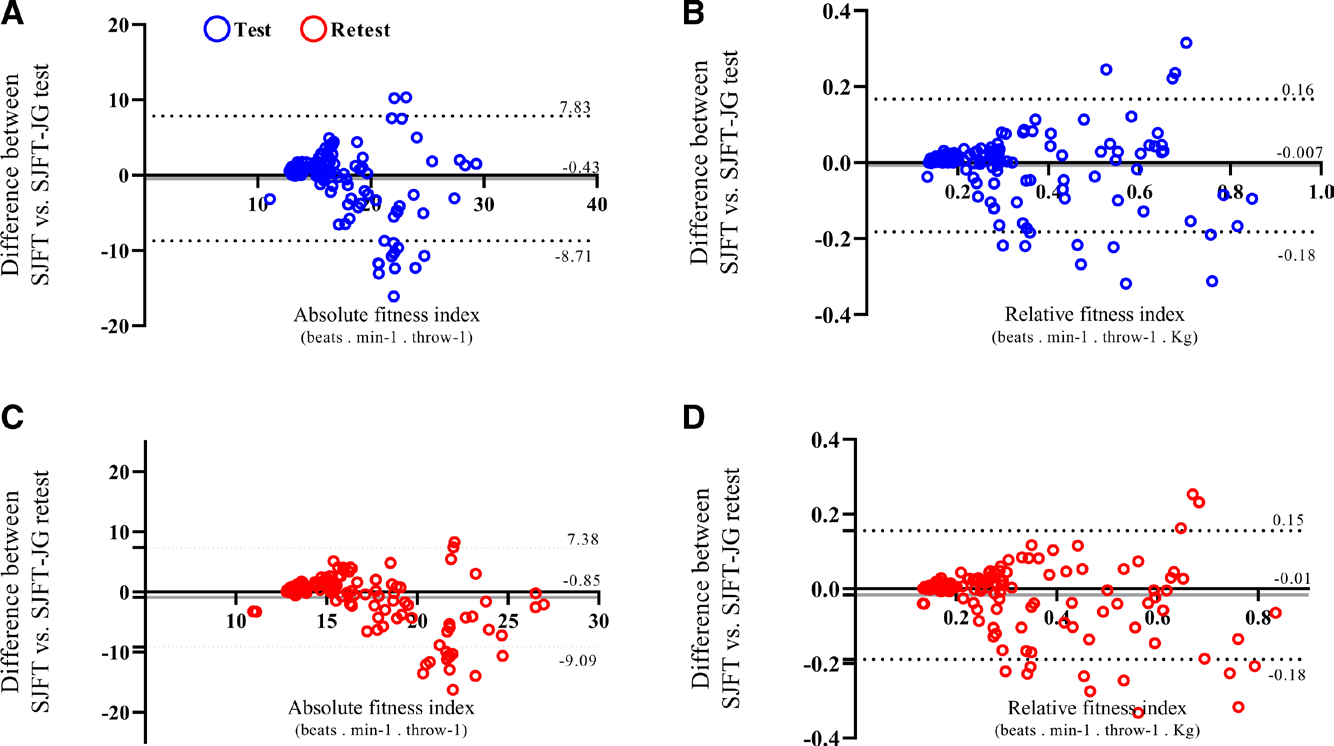
Differences between SJFT and SJFT-JG methods in the test and retest moments of this study. Data treatment: Bland–Altman plot. (Beats·min^-1^·throw^-1^) = (Beats per minute/number of throw), (Beats·min^-1^·throw^-1^·kg) = (Beats per minute/number of throw)/kilogram, SJFT = special Judo fit test, SJFT-JG = special Judo fitness test using the juji-gatame technique.

**Figure 5.**
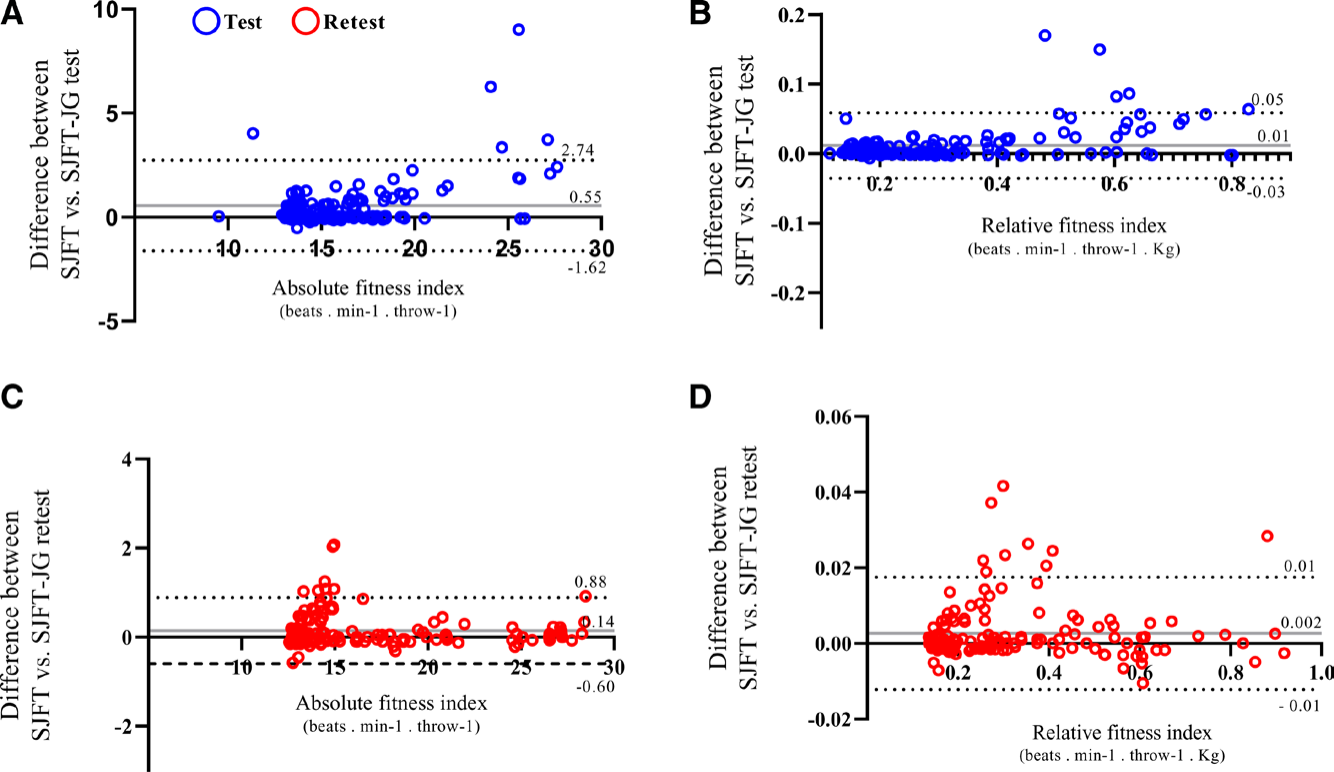
Intra-method differences in the test and retest moments of the present study considering the SJFT and SJFT-JG. Data treatment: Bland-Altman plot. (Beats/min/throw) = Beats per minute/number of throws, (beats/min/throw/kg) = (Beats per minute/number of throws)/kilogram, SJFT = special judo fitness test, SJFT-JG = special judo fitness test using the juji-gatame technique.

Concordance analyses between the SJFT and SJFT-JG methods indicated that the differences between test and retest moments were <1.0 for both absolute (Fig. 4A and C) and relative (Fig. 4B and D) fitness index. However, the upper and lower LoA values indicate considerable disagreement between the methods for absolute indexes (Fig. 4A and C), whereas for relative indexes, the LoA values are acceptable (between −5 and 5) for both test and retest moments (Fig. 4B and D). Intra-method concordance analyses (SJFT-test with SJFT-retest, SJFT-JG test with SJFT-JG retest) indicated that both methods are reproducible, showing differences <1.0 and acceptable LoA values (between −5 and 5) for both absolute (Fig. 5A and C) and relative (Fig. 5B and D) indexes, with smaller differences and LoA values for the SJFT-JG method compared to the SJFT method.

Significant ICC values were observed between tests and intra-tests (Table 2), considering both moments of the present study (test and retest).

**Table 2:** Intra-class correlation coefficient of the SJFT and SJFT-JG (fitness index) methods in relation to the test and retest moments of the present study.

Methods		ICC	(95% CI)	*P*-value
Test	Test	–	–	–
SJFT (Beats·min^-1^·throw^-1^)	SJFT-JG (Beats·min^-1^·throw^-1^)	0.698	0.583–0.781	**.01** *
SJFT (Beats·min^-1^·throw^-1^·kg)	SJFT-JG (Beats·min^-1^·throw^-1^·kg)	0.937	0.913–0.955	**.01** *
Retest	Retest	–	–	–
SJFT (Beats·min^-1^·throw^-1^)	SJFT-JG (Beats·min^-1^·throw^-1^)	0.661	0.532–0.755	**.03** *
SJFT (Beats·min^-1^·throw^-1^·kg)	SJFT-JG (Beats·min^-1^·throw^-1^·kg)	0.935	0.910–0.953	**.001** *
Test	Retest			
SJFT (Beats·min^-1^·throw^-1^)	SJFT-JG (Beats·min^-1^·throw^-1^)	0.690	0.572–0.775	**.02** *
SJFT (Beats·min^-1^·throw^-1^)	SJFT (Beats·min^-1^·throw^-1^)	0.972	0.962–0.980	**.001** *
SJFT-JG (Beats·min^-1^·throw^-1^)	SJFT-JG (Beats·min^-1^·throw^-1^)	`	0.870–0.999	**.001** *
SJFT (Beats·min^-1^·throw^-1^·kg)	SJFT-JG (Beats·min^-1^·throw^-1^·kg)	0.936	0.911–0.953	**.001** *
SJFT (Beats·min^-1^·throw^-1^·kg)	SJFT (Beats·min^-1^·throw^-1^·kg)	0.995	0.993–0.996	**.001** *
SJFT-JG (Beats·min^-1^·throw^-1^ kg)	SJFT-JG (Beats·min^-1^·throw^-1^·kg)	0.980	0.950–1.00	**.0001** *

Bold values indicate statistical significance (*P* < .05).

95% CI = 95% confidence interval, Beats·min^-1^·throw^-1^ = beats per minute/number of throw, Beats·min^-1^·throw^-1^·kg = Beats per minute/number of throw/kilogram, ICC = intra-class correlation coefficient, SJFT = special judô fit test, SJFT-JG = special judo fitness test using the juji-gatame technique.

* Statistically significant. Magnitude of ICC: absence: <0; poor: 0–0.19; weak: 0.20–0.39; moderate: 0.30–0.59; substantial: 0.60–0.79; almost complete: ≥0.80. Data treatment: Intra-class correlation coefficient.

When comparing the methods at the test and retest time points (intra-test comparisons), no significant differences were found in the absolute and relative fitness indices assessed by the SJFT and SJFT-JG (Table 3).

**Table 3:** Comparisons between SJFT and SJFT-JG methods for the test and retest moments of the study.

Moment	SJFT (Beats·min^-1^·throw^-1^)	*P*-value	ES	(95% CI ES)	Covariates				
					Age	PHV	Tanner	Training time (year)	Belt Color
					*P*-value				
Test	16.5 ± 3.6	.5	0.05	0.01; 0.10	.6	.9	.1	.7	.9
Retest	15.9 ± 3.1	–	–	–	–	–	–	–	–
	SJFT (Beats·min^-1^·throw^-1^·kg)	–	–	–	–	–	–	–	–
Test	0.3 ± 0.1	.7	0.04	0.00; 0.13	.9	.6	.7	.8	.9
Retest	0.2 ± 0.1	–	–	–	–	–	–	–	–
	SJFT-JG (Beats·min^-1^·throw^-1^)	–	–	–	–	–	–	–	–
Test	16.9 ± 3.9	.8	0.00	0.00; 0.05	.8	.8	.9	.6	.2
Retest	16.7 ± 5.0	–	–	–	–	–	–	–	–
	SJFT-J (Beats·min^-1^·throw^-1^·kg)	–	–	–	–	–	–	–	–
Test	0.3 ± 0.1	.7	0.01	0.00; 0.15	.7	.7	.1	.7	.3
Retest	0.3 ± 0.1	–	–	–	–	–	–	–	–

Data treatment: repeated measures ANOVA.

95% CI = 95 percent confidence interval, Beats·min^-1^·throw^-1^ = beats per minute/number of throw, Beats·min^-1^·throw^-1^·kg = beats per minute/number of throw/kilogram, ES = effect size, SJFT = special judô fit test, SJFT-JG = special judo fitness test using the juji-gatame technique.

**Figure 6.**
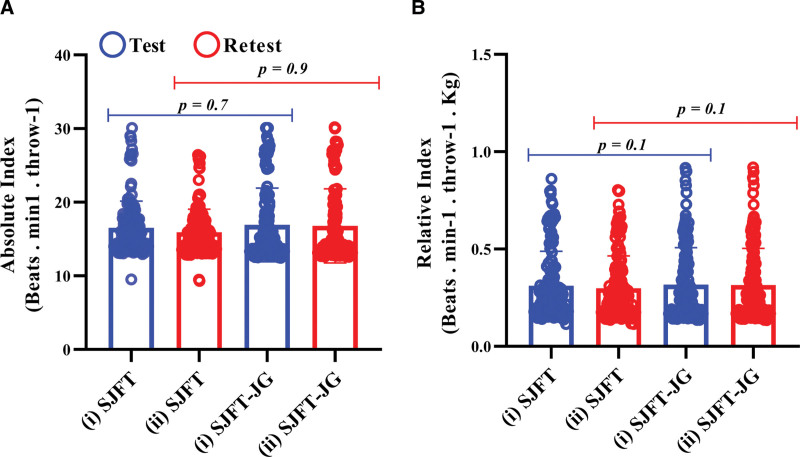
(A) Absolute index, and (B) relative index. (i): Test. (ii): Retest. Data treatment: Repeated measures ANOVA. (Beats·min^-1^·throw^-1^) = beats per minute/number of throw, (Beats·min^-1^·throw^-1^·kg) = (beats per minute/number of throw)/kilogram, SJFT = special judô fit test, SJFT-JG = special judo fitness test using the juji-gatame technique.

When comparing the methods, no significant differences were found in the absolute and relative fitness indices analyzed, suggesting that there are no meaningful differences between the conventional SJFT and the SJFT-JG (Fig. 6). Additionally, no significant effects were observed for the covariates age (P >.05), PHV (P >.05), Tanner stages (P >.05), training time (P >.05), or belt color (P >.05).

## 4. Discussion

This study investigated the applicability of the JG technique in the SJFT (SJFT-JG) as an alternative to assess the physical condition of grappling athletes. The results demonstrated that the SJFT-JG generates absolute and relative fitness indices that did not differ significantly from those obtained with the conventional SJFT. These findings support the initial hypothesis that the adaptation of the SJFT with the employment of the ippon-seoi-nage technique allows an effective measurement of physical condition, especially for athletes who prefer ground combat techniques.

### 4.1. Reliability and vof the SJFT-JG

The similarity in results between the SJFT and SJFT-JG suggests that the JG technique can be a valid tool for assessing grappling athlete’s performance. This is particularly relevant, given that many fighters specialize in ground techniques and may feel more comfortable and confident using their specific skills during the assessment.^[8,9]^ Furthermore, the use of a training dummy for the execution of the SJFT-JG eliminated concerns regarding injuries, allowing participants to focus on maximizing their performance without the risk of injuring a training partner.^[4]^ Additionally, the intra-class correlation coefficients (ICC) for the SJFT-JG were substantial to nearly complete (ICC ≥ 0.80), confirming the reproducibility of the SJFT-JG, in alignment with previous studies that validated the SJFT for other grappling modalities, such as wrestling^[7]^ and jiu-jitsu.^[22]^

Additionally, the SJFT-JG’s reliability is further supported by its results consistency across different testing sessions, as evidenced by the strong ICC values observed in both test and retest scenarios.^[20,23]^ This consistency is crucial for coaches and sports scientists who rely on reproducible data to make informed decisions about training programs and athlete performance.^[24–26]^ The SJFT-JG’s adaptability to different grappling styles like Brazilian Jiu-Jitsu and submission wrestling also highlights its versatility as a fitness assessment tool.^[8]^ This adaptability is particularly important in combat sports, where athletes often transition between different disciplines and require assessments that reflect their specific training needs.^[27]^

Furthermore, the agreement between the SJFT and SJFT-JG methods, particularly in relative indices, underscores the importance of considering individual differences in body mass and fitness levels when assessing athletes. This is especially relevant in combat sports, where weight classes and body composition play a significant role in performance.^[28]^ The SJFT-JG’s ability to provide reliable relative fitness indices makes it a valuable tool for coaches and sports scientists working with athletes across different weight categories. Future studies could explore the relationship between body composition and SJFT-JG performance to further refine its application in athlete assessment.^[8]^

### 4.2. Physiological demands and agreement between methods

The results of the agreement between methods indicated that, although there are discrepancies in absolute indices, the relative indices showed acceptable limits of agreement (LoA between −5 and 5). This suggests that while the SJFT may be more sensitive to variations in absolute performance, the SJFT-JG is effective in assessing the relative physical condition of athletes.^[23]^ The Bland-Altman analysis reinforced this agreement,^[29]^ highlighting that the SJFT-JG can be used interchangeably with the traditional SJFT, especially when assessing athletes who are more proficient in ground techniques. Additionally, the SJFT-JG elicited HR responses and fitness indices similar to those of the traditional SJFT, indicating that both tests impose comparable physiological stress, consistent with the design of the SJFT, which simulates the effort/rest ratios (3:1)^[1,2]^ observed in judo and other grappling sports.^[6–8]^

### 4.3. Practical applications

The SJFT-JG is a valuable tool for sports professionals working with grappling athletes, and allows the assessment of physical condition respecting the specificities of ground techniques. It is recommended that professionals conduct the test in a controlled environment and, if possible, record the execution of the test on video; this will assist in monitoring the number of correct executions of the JG technique. It is also necessary to record the HR immediately after the effort and 1 minute later in order to calculate the absolute and relative fitness indices. This approach not only facilitates the identification of areas for improvement but also allows for continuous monitoring of athletes’ progress, both in junior and adult categories, ensuring that training programs are adjusted according to individual needs and levels of biological maturation (in the case of junior athletes). Furthermore, the use of the SJFT-JG in youth sports development programs can help create a more inclusive and motivating assessment environment, as junior athletes will feel more comfortable using their specific skills, promoting more effective and targeted training throughout their sports careers.

### 4.4. Limitations and suggestions for future studies

Although the SJFT-JG has demonstrated reliability and validity, some limitations must be acknowledged. The study focused on male athletes; future research should include female participants to ensure the generalization of findings. Additionally, the inclusion of different grappling styles could further enhance the applicability of the results. Finally, the long-term effects of using the SJFT-JG in training programs should be investigated to assess its impact on performance and injury prevention.

## 5. Conclusion

Based on the results of this study, we conclude that the JG technique can be effectively used in the SJFT to assess the physical condition of grappling athletes across different age groups, providing a viable alternative to the traditional method that utilizes the ippon-seoi-nage. This adaptation not only meets the needs of athletes who specialize in ground combat but also contributes to a broader understanding of physical performance across various combat sports. The validation of the SJFT-JG opens new possibilities for personalized athlete assessments, promoting more effective and targeted training.

## Acknowledgments

For your support and encouragement for the development of this academic article, we thank the Federal University of Rio Grande do Norte (UFRN), the Physical Activity and Health (AFISA) research base, the Research Group on Sports and Human Performance (GPDEH), the Dalton Cunha blood center in the city of Natal/Brazil. The National Council for Scientific Development (CNPQ) and the Higher Education Personnel Improvement Coordination (CAPES).

## Author contributions

**Conceptualization:** Paulo Francisco de Almeida-Neto, Alexandre Bulhões-Correia, Halil İbrahim Ceylan, Breno Guilherme de Araújo Tinôco Cabral.

**Data curation:** Paulo Francisco de Almeida-Neto, Alexandre Bulhões-Correia, José Carlos Gomes da Silva.

**Formal analysis:** Paulo Francisco de Almeida-Neto, Alexandre Bulhões-Correia, Roberto Felipe Câmara Rocha, Iago Medeiros da Silva.

**Investigation:** Alexandre Bulhões-Correia, Roberto Felipe Câmara Rocha, Iago Medeiros da Silva.

**Methodology:** Paulo Francisco de Almeida-Neto.

**Supervision:** Gilmara Gomes de Assis, Valentina Stefanica, Breno Guilherme de Araújo Tinôco Cabral.

**Writing – original draft:** Paulo Francisco de Almeida-Neto, Alexandre Bulhões-Correia, Gilmara Gomes de Assis, Halil İbrahim Ceylan, Roberto Felipe Câmara Rocha, Iago Medeiros da Silva, José Carlos Gomes da Silva, Paulo Moreira Silva Dantas, Valentina Stefanica, Breno Guilherme de Araújo Tinôco Cabral.

**Writing – review & editing:** Paulo Francisco de Almeida-Neto, Alexandre Bulhões-Correia, Halil İbrahim Ceylan, Roberto Felipe Câmara Rocha, Iago Medeiros da Silva, José Carlos Gomes da Silva, Paulo Moreira Silva Dantas, Valentina Stefanica, Breno Guilherme de Araújo Tinôco Cabral.
